# The Ethical and Societal Considerations for the Rise of Artificial Intelligence and Big Data in Ophthalmology

**DOI:** 10.3389/fmed.2022.845522

**Published:** 2022-06-28

**Authors:** T. Y. Alvin Liu, Jo-Hsuan Wu

**Affiliations:** ^1^Wilmer Eye Institute, Johns Hopkins University, Baltimore, MD, United States; ^2^Shiley Eye Institute and Viterbi Family Department of Ophthalmology, University of California, San Diego, La Jolla, CA, United States

**Keywords:** ethics, bias, artificial intelligence, fairness, privacy

## Abstract

Medical specialties with access to a large amount of imaging data, such as ophthalmology, have been at the forefront of the artificial intelligence (AI) revolution in medicine, driven by deep learning (DL) and big data. With the rise of AI and big data, there has also been increasing concern on the issues of bias and privacy, which can be partially addressed by low-shot learning, generative DL, federated learning and a “model-to-data” approach, as demonstrated by various groups of investigators in ophthalmology. However, to adequately tackle the ethical and societal challenges associated with the rise of AI in ophthalmology, a more comprehensive approach is preferable. Specifically, AI should be viewed as sociotechnical, meaning this technology shapes, and is shaped by social phenomena.

## Introduction

The rise of artificial intelligence (AI) and big data has been hailed as the 4th Industrial Revolution. Recent advancement in AI, in the form of deep learning (DL) which is a subtype of machine learning (ML), and improvement in hardware such as graphic processing units (GPU), have propelled medical AI applications to the forefront of the public discourse. This is because DL has been shown to be on par with human experts in analyzing medical images across different specialties, especially in medical specialties that interact with and have access to a large number of images, such as dermatology, radiology, and ophthalmology (1–10). In addition, “super-human” feats achieved by DL, such as the robust prediction of age, gender, blood pressure and smoking status of a person from a color fundus photograph alone (11), have captured the public's imagination and sparked a debate on the role and impact of AI on medicine.

Ophthalmology, being at the forefront of this AI revolution in medicine, is well-positioned to actively participate in and be a thought-leader on the societal implications for the rise of AI and big data in medicine. In the following perspective piece, we will highlight the ethical controversies and considerations from an ophthalmological perspective. The two major concerns regarding the rise of AI in medicine and ophthalmology center on bias and privacy.

## Discussion

### Bias and Fairness

AI has the potential to entrench, or even exacerbate, existing biases in the healthcare system *via* unfair recommendations or decision-making. Fairness can be defined as “the absence of any prejudice or favoritism toward an individual or a group based on their inherent or acquired characteristics” (12). A prominent example of a medical AI algorithm providing unfair recommendations and exacerbating biases was highlighted by a study by Obermeyer at al. (13) showing that an AI algorithm systematically biased against Black patients, by erroneously using previous health costs as a proxy for predicting health needs and illness severity.

Bias in the training data is one of the most common reasons for a ML algorithm to produce unfair downstream predictions or recommendations. Many types of bias in ML exist. A comprehensive discussion of the different kinds of bias is beyond the scope of the current paper, but is nicely summarized here (14, 15). Specifically, within the context of ophthalmology DL studies, imbalance in training images is a common, yet addressable, reason that can lead to biases against a patient subgroup, such as patients of a certain race. For example, the AREDS image dataset (16), generated from a landmark longitudinal clinical trial and used in numerous important ophthalmology DL studies, was derived primarily from Caucasian patients (about 96% of participants). While age-related macular degeneration (AMD) is more prevalent in Caucasian patients (prevalence of 5.4% vs. 4.2% in Hispanic, 2.4% in Black and 4.6% in Asian) (17–19), the difference in prevalence on a population level does not explain fully the extreme imbalance in the AREDS dataset. Additional factors, such as unequal access to or interest in participating in clinical trials, likely also played a role.

However, such imbalance in training data can be addressed in three different ways. First, patient recruitment in prospective studies can be planned to ensure equal enrollment numbers for different pre-specified patient subgroups, e.g., based on sex, age, race, ethnicity, socioeconomic status and disease severity, etc. Second, if the recruitment of a certain patient subgroup is limited by practicality or natural prevalence of the disease, e.g., Black patients with AMD, then low-shot DL can be attempted. Low-shot DL, in contrast to traditional DL which requires a large amount of data for training, can be trained with relatively few samples (20), and can outperform traditional DL approaches when the available training dataset is small (5). Third, the patient subgroup that is under-represented in the training samples can be augmented by generative DL, a DL technique that can generate synthetic data. It has been shown that retinal images, created by generative DL, can be used to train a robust DL system for AMD classification (21). Specifically, in the context of DL-based detection of referable diabetic retinopathy, generative DL has been used to increase the training image samples of an under-represented patient subgroup and has been shown to decrease the bias against that particular under-represented patient subgroup during testing (22).

In addition to addressing the data distribution, the model itself can be fine-tuned to improve fairness. For example, instead of minimizing the average error across all statistics, we could aim to minimize the maximum error of a subset of statistics as evaluated across different demographic groups of interest.

A recent scoping review on digital health solutions (23) found that AI health applications generally lacked vigorous pragmatic prospective real-world validations. Addressing training data imbalance during model development should produce more generalizable ophthalmic AI applications that perform more robustly in real-world validations.

### Privacy

DL models typically require a large amount of data for training, and the rise of DL in ophthalmology coincided with the rise of big data, both in the form of images and tabular data. The training and testing of DL models often involve combining ophthalmic images from different sources, and there is increasing concern that such transfer of data represents an unacceptable risk of privacy breach, especially since fundus images are now considered protected health information.

Such concerns can be addressed in two ways: federated learning and differential privacy. The training of DL models can be facilitated by federated learning, which allows model training in a decentralized fashion, takes advantage of the data heterogeneity from disparate sources, and does not require actual transfer of data between the sources (24). This approach has been successfully implemented in the context of retinal microvasculature segmentation and referable diabetic retinopathy detection on optical coherence tomography (OCT) and OCT angiography images. The authors demonstrated that a federated learning approach achieved similar results as a traditional centralized learning approach (25). Similarly, instead of transferring data to train a DL model, the model itself can be “brought” to the data for retraining. This concept has been successfully demonstrated in the context of DL-based intraretinal fluid segmentation on OCT images, in which the parameters of a pre-trained DL model were frozen, transferred to and retrained at a different institution. The authors showed that such a “model-to-data” work flow could update a model and improve the model's performance, without the transfer of actual data (26).

Besides image databases, ophthalmology is also at the forefront of establishing massive tabular databases. The Intelligent Research in Sight (IRIS) Registry, spearheaded by the American Academy of Ophthalmology, is the largest specialty database in all of medicine in the world. The data collected to date has been invaluable, and led to numerous new insights and publications. Without a question, the IRIS Registry will be indispensable in developing the next-generation predictive ML algorithms. The data collected in IRIS is first de-identified, before being distributed to researchers. Traditional data de-identification methods include complete removal of all unique identifiers or coarsening of the original dataset. Data coarsening is achieved by providing the exact values of only a subset of the original sample and thus creating an incomplete dataset (27, 28). What remains to be seen is whether traditional data de-identification methods will be sufficient for protecting the privacy of data in the IRIS registry or similar tabular databases. Traditional de-identification methods are vulnerable to linkage and other re-identification attacks, in which third parties correlate the supposedly anonymized data with unanticipated sources of auxiliary information to learn sensitive information about data participants. Examples of de-identification failure include the re-identification of “anonymized” hospital records released by Massachusetts' Group Insurance Commission and the re-identification of Netflix users' movie reviews from a dataset released as part of a ML challenge that Netflix hosted in 2006. A promising avenue of research is the application of differential privacy to large ophthalmic databases, such as IRIS.

Differential privacy is the only principled solution for releasing aggregate information about a statistical database, with provable guarantees that no information attributable to any individual in the dataset will be revealed. Briefly, differential privacy employs randomization to guarantee that the log odds ratio of any output of the analysis is bounded by and compared to a counterfactual world, in which any given participant has been entirely removed from the dataset, thereby formally limiting what inferences an arbitrarily well-informed observer can make about the data of any single participant (29). By definition, differential privacy prevents membership inference attacks as discussed above and provides a general umbrella of protection. However, the exact methods to create a differentially private dataset of unstructured data, e.g., ophthalmic images, are not currently available. This a major limitation of differential privacy as most recent advances in ML applications to ophthalmology have been in DL applications to ophthalmic images.

Finally, next-generation data infrastructure, specifically geared toward big data, ML and data privacy, is being developed, and a cutting-edge example is swarm learning. Swarm learning (30) is a decentralized data infrastructure that uses blockchain technology to ensure peer-to-peer data security. In contrast to federated learning which still requires a central parameter server, swarm learning is completely decentralized and, in addition, could inherit and be compatible with aforementioned differential privacy algorithms.

## Conclusion

We are in the midst of the 4th Industrial Revolution, and ophthalmology has been at the forefront of the rise in AI/ML/DL and big data in medicine, and encountered various ethical and societal implications of this trend. While the concerns surrounding bias, fairness and privacy can be partially addressed by the strategies outlined above, a more comprehensive approach is preferable. This shift in mentality is best demonstrated by a recently announced special funding opportunity that was offered by the National Institute of Health as part of the Bridge2AI Common Fund^1^. The funding opportunity aims to produce Data Generation Projects that prospectively curate AI/ML ready data based on ethical principles. Multi-disciplinary teams, comprised of physicians, computer scientists and ethicists, are expected to promote a culture of ethical inquiry and consider ethical issues throughout the entire lifecycle of the project. Such an approach is grounded in the emerging view that AI is a sociotechnical issue: that is, AI shapes, and is shaped by social phenomena. The acknowledgment that the successful application of AI to medicine hinges on the holistic tackling of the associated ethical and societal implications is indeed a huge step forward, and we predict ophthalmologists in particular will play an important role in this regard in the years to come.

## Data Availability Statement

The original contributions presented in the study are included in the article/supplementary materials, further inquiries can be directed to the corresponding author.

## Author Contributions

All authors listed have made a substantial, direct, and intellectual contribution to the work and approved it for publication.

## Conflict of Interest

The authors declare that the research was conducted in the absence of any commercial or financial relationships that could be construed as a potential conflict of interest.

## Publisher's Note

All claims expressed in this article are solely those of the authors and do not necessarily represent those of their affiliated organizations, or those of the publisher, the editors and the reviewers. Any product that may be evaluated in this article, or claim that may be made by its manufacturer, is not guaranteed or endorsed by the publisher.

## Footnotes

^1^ https://commonfund.nih.gov/bridge2ai
